# General anesthesia with S-ketamine improves the early recovery and cognitive function in patients undergoing modified radical mastectomy: a prospective randomized controlled trial

**DOI:** 10.1186/s12871-023-02161-6

**Published:** 2023-06-20

**Authors:** Junxia Zhang, Danting Jia, Wenbin Li, Xiaohui Li, Qian Ma, Xuexin Chen

**Affiliations:** Department of Anaesthesia and Perioperative Medicine, Cancer Hospital, General Hospital of Ningxia Medical University, No. 804 Shengli South Street, Xingqing District, Yinchuan, Ningxia, 750004 People’s Republic of China

**Keywords:** S-ketamine, Quality of recovery, Cognitive function, Breast cancer, Modified radical mastectomy

## Abstract

**Background:**

Postoperative cognitive dysfunction (POCD) is a common postoperative disorder that is frequently observed after general anesthesia, which seriously threatens the quality of patients’ life. Existing studies have demonstrated that S-ketamine plays an important role in improving neuroinflammation. This trial aimed to explore the effects of S-ketamine on quality of recovery and cognitive function in patients following modified radical mastectomy (MRM).

**Methods:**

Ninety patients aged 45 to 70 years with ASA grades of I or II, who underwent MRM, were selected. Patients were randomly assigned to the S-ketamine or control group. In the S-ketamine group, patients were induced with S-ketamine instead of sufentanil and maintained with S-ketamine and remifentanil. In the control group, patients were induced with sufentanil and maintained with remifentanil. The primary outcome was the Mini-Mental State Examination (MMSE) and Quality of Recovery-15 (QoR-15) score. Secondary outcomes including visual analog scale (VAS) score, cumulative propofol and opioids consumption, post anesthesia care unit (PACU) recovery time, occurrence of remedial analgesia, postoperative nausea and vomiting (PONV), other adverse events, as well as patient satisfaction.

**Results:**

The global QoR-15 scores at postoperative day 1 (POD1) were significantly higher in the S-ketamine group than in the control group (124 [119.5–128.0] vs. 119 [114.0–123.5], *P* = 0.002), with a median difference of 5 points (95% confidence interval [CI] [-8 to -2]). Similarly, the global QoR-15 scores at postoperative day 2 (POD2) in the S-ketamine group were significantly higher than in the control group (140.0 [133.0–145.0] vs. 132.0 [126.5–141.5], *P* = 0.004). In addition, among the five subcomponents of the 15-item scale, S-ketamine group had a higher score in terms of physical comfort, pain, and emotional state both at POD1 and POD2. In terms of MMSE score, S-ketamine could promote the recovery of postoperative cognitive function at POD1, but not at POD2. Furthermore, the consumption of opioids, VAS score, and remedial analgesia in the S-ketamine group decreased significantly.

**Conclusions:**

Collectively, our findings support that general anesthesia with S-ketamine as a potential strategy showed high safety and could not only improve the quality of recovery mainly through improving pain, physical comfort, and emotional state but also promote the recovery of cognitive function on POD1 in patients undergoing MRM.

**Trial registration:**

The study was registered in the Chinese Clinical Trial Registry (registration No:ChiCTR2200057226, Date of registration: 04/03/2022).

## Background

Breast cancer (BC) is one of the malignant cancers with the highest incidence in women worldwide [1]. To date, modified radical mastectomy (MRM) remains the main therapy for BC, which is a painful procedure and inevitably accompanied by breast loss and body shape change. Accordingly, patients with BC may experience surgical trauma and severe postoperative pain, coupled with psychological trauma and aggravated emotional and cognitive problems [2]. Postoperative cognitive dysfunction (POCD), characterized by impairments in memory, attention, consciousness, and sleep cycle, is a common complication that is frequently observed after general anesthesia [3]. All of these clinically relevant complications can be seriously detrimental to individual health, decrease the quality of perioperative rehabilitation and pose a threat to the long-term prognosis of patients with BC [4], which compelled us to pay more attention to patients’ postoperative recovery. Therefore, it is crucial to develop a potential protocol to accelerate patients’ recovery after BC surgery.

S-ketamine, an N-methyl-D-aspartate (NMDA) receptor antagonist, a novel antidepressant drug, can quickly eliminate patient’s depressive state, alleviate anxiety symptoms, and benefit for both physical and mental health of patient [5, 6]. Most importantly, accumulating evidence has demonstrated that S-ketamine presents a great safety profile and reliability as part of multimodal analgesia regimen. And S-ketamine is considerably more superior than other intravenous sedatives, for its application can not only stabilize perioperative hemodynamic fluctuations, relieve perioperative stress and inflammatory response, and result in fewer side effects, but also been proved has neuroprotective effect [7]. Collectively, S-ketamine have been widely used as adjunctive therapy to general anesthesia to achieve optimal pain management and accelerate emotional recovery, which deserves to be promoted in clinical anesthesia management [8–10].

Nevertheless, research on S-ketamine mainly focuses on antidepressant therapy, and few studies have attached importance to patients’ perioperative rehabilitation and cognitive function so far, which is vitally important to patient prognosis. Accordingly, this trial aimed to explore the effects of general anesthesia with S-ketamine on quality of recovery and cognitive function in patient with BC. We hypothesized that general anesthesia with S-ketamine could accelerate the perioperative quality of recovery and improve the cognitive function rehabilitation in patients following an MRM.

## Methods

### Ethics approval

This trial was approved by the Ethics Committee of the General Hospital of Ningxia Medical University in February 2022 (approval No.: KYLL-2022-0056) and was registered prospectively on the Chinese Clinical Trial Registry (ChiCTR) (http://www.chictr.org.cn) on 04/03/2022 (registration No.: ChiCTR2200057226). Written informed consent was obtained from all subjects participating in the trial, and the manuscript adheres to the applicable CONSORT guidelines.

### Design

This study was a prospective randomized controlled trial performed at the General Hospital of Ningxia Medical University from 10/03/2022 to 28/12/2022. 90 female patients aged 45–75 years, with American Society of Anesthesiologists (ASA) physical status I or II, undergoing MRM were enrolled in this study. MRM was defined as the removal of the entire breast, including the skin, areola, nipple, and most axillary lymph nodes with preservation of the pectoralis major and minor muscles. Patients complicated with severe diseases including severe heart, lung, liver, kidney, or endocrine diseases, cognitive impairment, currently on sedative opioids or sleep-aid medication (benzodiazepines), use of anti-depression medications (such as: tricyclic, tetracyclic antidepressants or selective serotonin reuptake inhibitors) were excluded. Using a computer-generated random number table, all participants were randomly allocated to either the control or the S-ketamine group following a 1:1 ratio. The group allocation was concealed in sealed and opaque envelopes, which was opened by the anesthesiologist after the patient entered the operation room. The study medications were prepared by an attending anesthetist so that the designated researcher was blinded to the intervention.

### Interventions

Patients in the S-ketamine group received 0.5 mg/kg S-ketamine instead of sufentanil for induction and were maintained with remifentanil and S-ketamine (followed by 0.5 mg·kg^− 1^·h^− 1^ continuous infusion from induction of anesthesia to the 30 min before the end of surgery). In the control group, patients were induced with sufentanil and maintained with remifentanil, but they did not receive S-ketamine during the process of anesthesia. Dose of S-ketamine in the current study based on Lavender’ study, they suggested an induction dose of S-ketamine is 0.5–1 mg/kg, and the maintenance dose is 0.5–3 mg/kg [11]. Therefore, the doses in the present study were identified as within a safe dose range [12].

### Anesthesia

All enrolled patients were injected with 1 mg of penehyclidine hydrochloride 30 min before surgery. Routine monitoring including electrocardiography, heart rate, non-invasive blood pressure, pulse oximetry, bispectral index (BIS), and TOF-Watch-SX (Organon, Oss, The Netherlands) were applied. All patients received total intravenous anesthesia. The anesthesia protocols in the two groups were as follows: Patients in the control group were induced with midazolam (0.05 mg/kg), sufentanil (0.3 µg/kg), propofol (2.0 mg/kg), and rocuronium (0.8 mg/kg) and were maintained with a target-controlled infusion (TCI) of propofol (4–6 mg·kg^− 1^·h^− 1^) and remifentanil (0.2–0.6 ug·kg^− 1^·min ^− 1^). Patients in the S-ketamine group were induced with midazolam (0.05 mg/kg), S-ketamine (0.5 mg/kg), propofol (2.0 mg/kg), and rocuronium (0.8 mg/kg) and maintained with a TCI of propofol (4–6 mg·kg^− 1^·h^− 1^), remifentanil (0.2–0.6 ug·kg^− 1^·min ^− 1^), and S-ketamine (0.5 mg·kg^− 1^·h^− 1^). The anesthetic administration rate was adjusted to the study protocol’s maintenance dose aiming for a BIS of 40–60 and mean arterial pressure (MAP) within 20% of the preoperative baseline values. After surgery all patients were transferred to the post-anesthesia care unit (PACU) for recovery.

### Outcome measures

The primary outcome was the global Quality of Recovery-15 (QoR-15) and Mini-Mental State Examination (MMSE) score measured at preoperatively (Pre), postoperative day 1 (POD1) and day 2 (POD2). The QoR-15 is a recovery-specific questionnaire that contains five dimensions: physical comfort, physical independence, psychological support, emotional state and pain, each of which is an 11-point numerical rating scale. Scores ranged from 0 to 150, with higher scores manifesting a more favorable health status [13]. The MMSE used in this study is a 30-question assessment, which reflected the cognitive function from the following aspects: immediate memory, time-oriented power, place-oriented power, delayed memory, language, visual space, attention, and calculation power. MMSE has a maximum total score of 30, with higher scores manifesting a better cognitive performance [14].

The secondary outcomes including postoperative VAS score, perioperative cumulative sufentanil and remifentanil consumption, the length of PACU stay, adverse events, occurrence of remedial analgesia, and patient satisfaction score. An 11-point VAS score was used to evaluate patient postoperative pain intensity at the end of surgery (T0) and 4 h (T1), 6 h (T2), 24 h (T3), and 48 h (T4) after surgery. All patients included in this study underwent unified perioperative pain management. Postoperative pain was assessed using visual analog scale (VAS), every 30 min until the first analgesic given, and 4 hourly for the next 48 h. Rescue analgesia was given in the form of injection diclofenac sodium 75 mg intramuscular when VAS score was more than 3. An 11-point Likert scale (range: 0–10, 0 indicates “entirely unsatisfied”, 10 indicates “fully satisfied.”) was used to assess patient satisfaction at postoperative day 2. In addition, adverse reactions, including psychotomimetic side effects (hallucinations or nightmares), dizziness, agitation, postoperative nausea and vomiting (PONV), and other adverse reactions were assessed up to 48 h postoperatively. The investigators responsible for assessing all the above results were unaware of the patients’ group assignment.

### Statistical analysis

Statistical analysis was performed using a statistical package for social sciences (SPSS, version 27.0, IBM Corporation, Armonk, NY, USA). The Kolmogorov–Smirnov test was used to evaluate the normality of data distribution. Parametric variables are recorded as mean ± standard and comparisons were made using the two-sample independent t-test. Continuous variables were analyzed using the Mann-Whitney U test and reported as median (interquartile range - IQR). Values presented as number (percentage, %) and comparisons were made using the chi-square test or Fisher’s exact test. The Hodges-Lehmann estimator was used to calculate the 95% confidence interval (CI) for the median difference. GraphPad Prism 8.0 (GraphPad Software, CA, USA) were used to draw all figures in this study. A p value < 0.05 was identified as statistically significant differences.

According to previous studies, an 8-point difference for the QoR-15 score signifies a clinically significant improvement [15]. On the basis of our preliminary data, the variability standard deviation of QoR-15 scores 24 h after BC surgery was 13. A sample size of 36 patients (without dropout) in each group was required to achieve a power of 80% with an alpha error of 0.05. Allowing for dropouts, we therefore planned to recruit 45 patients in per group with a total of 90 patients.

## Results

**Fig. 1:**
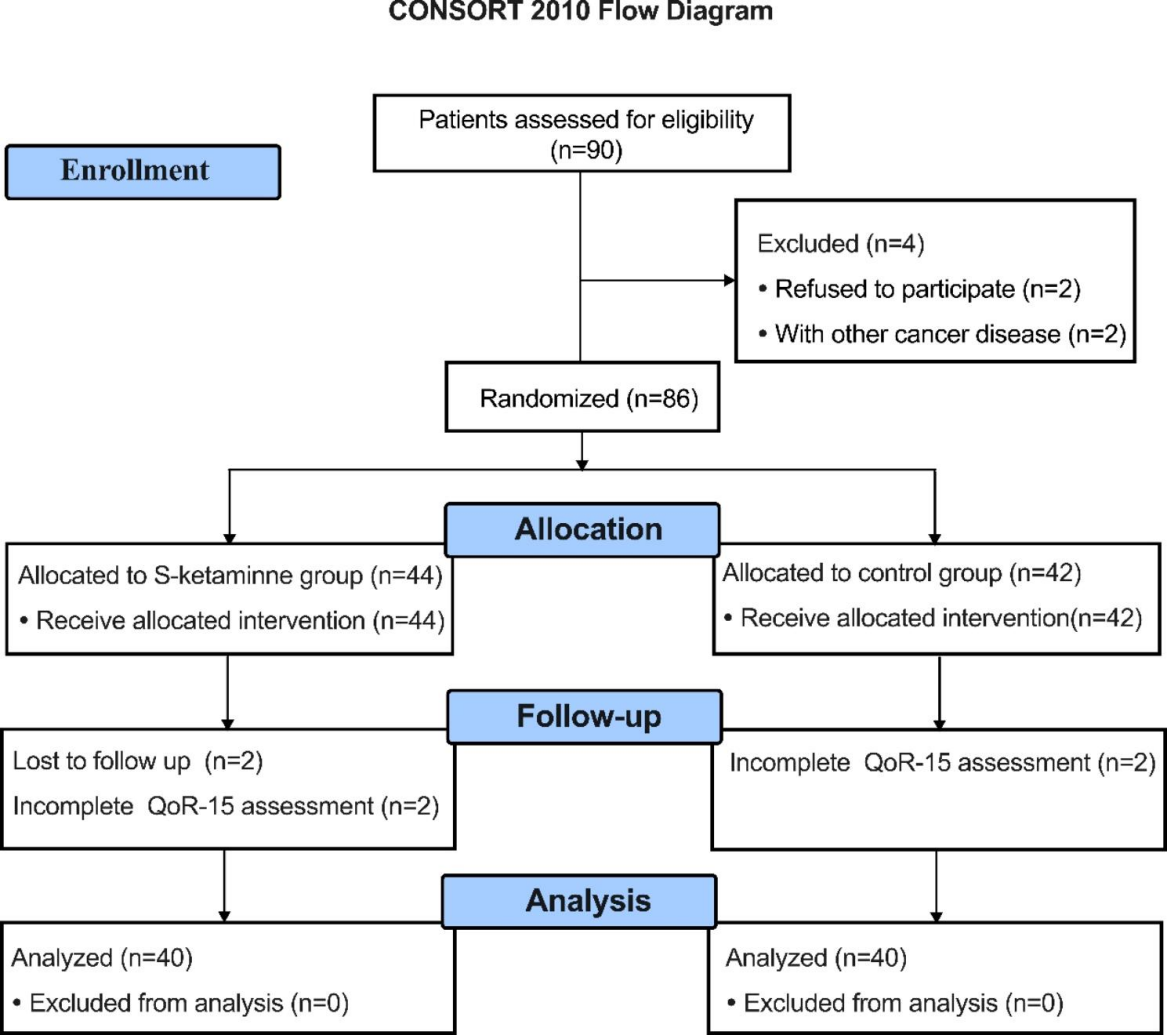
Consolidated Standards of Reporting Trials (CONSORT) flowchart describing patients progress through the study

From March 2022 to December 2022, 90 patients were initially evaluated for eligibility, of whom four patients met the exclusion criteria. Thus, 86 patients were randomized and received the study intervention. Later, four participants in S-ketamine group, two participants in control group were excluded because they were lost to follow-up or had incomplete postoperative QoR-15 assessment. Consequently, 80 patients were analyzed, with 40 patients in each group. The details of patient selection are illustrated in a flow chart (Fig. 1).

Patient characteristics and perioperative details of both groups are detailed in Table 1. Patient demographic data, preoperative QoR-15 scores, the duration of anesthesia and surgery were well balanced between the groups (*P* > 0.05). Given the strong sedative and analgesic effects of S-ketamine, the present study showed that the cumulative consumption of remifentanil, sufentanil and propofol were significantly decreased in the S-ketamine group when compared with the control group (*P* < 0.001) (Table 1).

**Table 1:** Patients’ demographic and perioperative characteristics (n = 40 in each group)

	Control Group	S-ketamine Group	*t/Z /X2*	*P*-value
Age (years)	54.9 ± 9.9	52.6 ± 10.9	0.963	0.338
BMI (kg/m^2^)	23.7 (22.5–25.9)	23.3 (22.2–24.2)	-1.338	0.181
ASA (I/II)	8/32	10/30	0.287	0.592
Menopause (%)	24.0 (60.0%)	20.0 (50.0%)	0.808	0.369
Duration of anesthesia (min)	118.5 ± 14.3	115.9 ± 17.4	0.722	0.472
Duration of surgery (min)	107.5 ± 23.6	101.0 ± 13.7	1.524	0.132
Remifentanil (mg)	1.5 ± 0.4	0.9 ± 0.3	6.549	< 0.001*
Sufentanil (ug)	18.0 (18.0–19.0)	0.0 (0.0–0.0)	-8.349	0.000*
Propofol (mg)	315.0 (270.0–340.0)	265.0 (205.0–300.0)	-3.159	0.002*
Preoperative QoR-15 score	141.5 (139.5–144.0)	142.0 (138.5–146.0)	1.222	0.222

Data are presented as median (interquartile range), mean ± SD or number (percentage). *P < 0.05, compared with control group. Abbreviations: ASA, American Society of Anesthesiologists; BMI, Body Mass Index; QoR-15, 15-item quality of recovery questionnaire

**Fig. 2:**
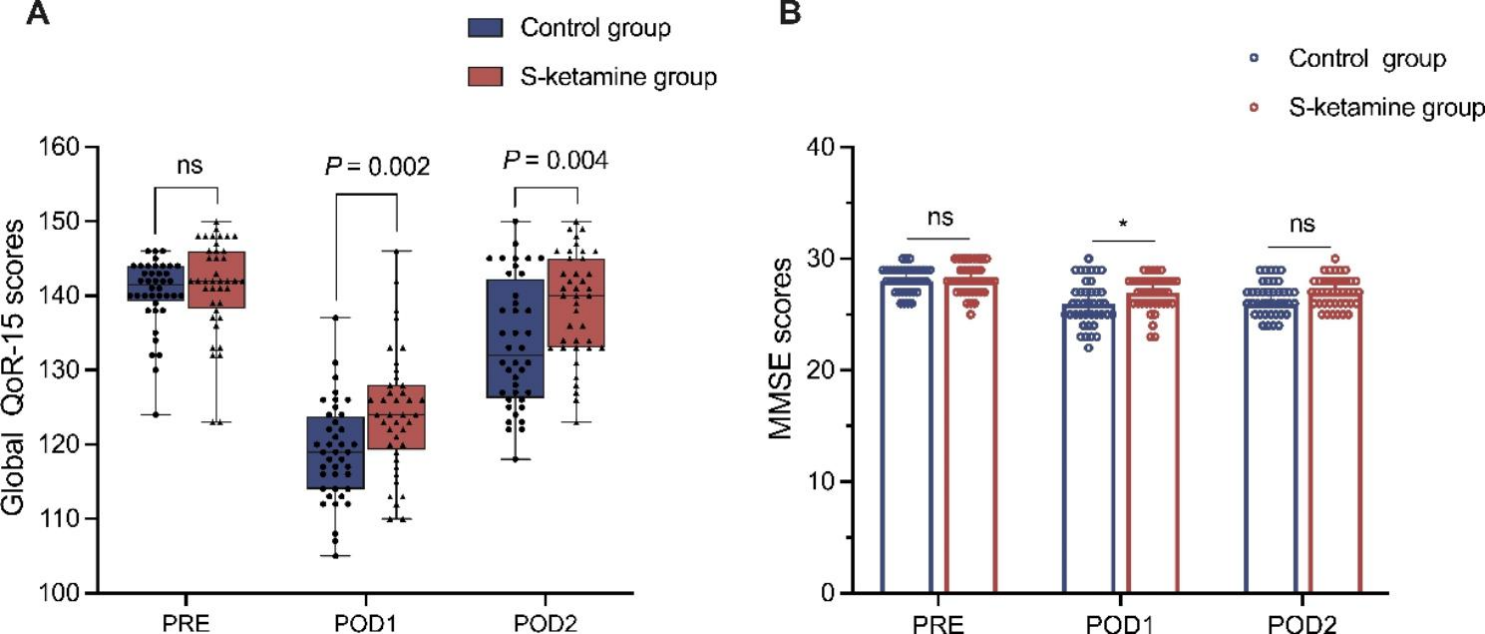
Comparison of the global QoR-15 scores of S-ketamine and propofol group at PRE, POD1 and POD2 (**A**); Comparison of the mini-mental state examination (MMSE) between the two groups (**B**). Data were expressed as median (horizontal bar), interquartile range (box), the maximum and minimum values (upper and lower edges) and the outliers (circles) in Fig. 2A; Data are presented as median (interquartile range) in Fig. 2B. ns, no significance; *P < 0.05, compared with control group

The global QoR-15 scores and five different subcomponent between the two groups at POD1 and POD2 are reported as median (IQR) in Table 2. Since 48 h after surgery is a pivotal time for patients’ recovery, therefore, we evaluated the QoR-15 scores of all patients both at POD1 and POD2. The global QoR-15 scores at POD1 were significantly higher in the S-ketamine group when compared with the control group (median: 124, IQR: 119.5–128.0 vs. median: 119, IQR: 114.0–123.5; 95%CI: -8 to -2, *P* = 0.002). Similarity, the global QoR-15 scores at POD2 were also higher in the S-ketamine group than in the control group (median: 140.0, IQR: 133.0–145.0 vs. median: 132.0, IQR: 126.5–141.5; 95% CI: -10 to -2, *P* = 0.004) (Table 2; Fig. 2A). The QoR-15 scale consists of a total of five components, each of which was analyzed separately. Among the five subdimensions of the QoR-15 scale, scores for pain, physical comfort, and emotional state at POD1 and POD2 were significantly higher in the S-ketamine group (*P* < 0.05), which indicated that S-ketamine can effectively control patients’ postoperative pain, relieve their negative emotions, and improve patients’ physical comfort. However, the subdimension scores for physical independence and psychological support both at POD1 and POD2 was comparable between the two groups (*P* > 0.05) (Table 2).

**Table 2:** Postoperative QoR-15 score (n = 40 in each group)

	Control Group	S-ketamine Group	Media Difference (95%CI)	*P*-value
Global QoR-15				
POD1	119.0 (114.0–123.5)	124.0 (119.5–128.0)	-5 (-8 to -2)	0.002*
POD2	132.0 (126.5–141.5)	140.0 (133.0–145.0)	-6 (-10 to -2)	0.004*
**QoR-15 dimensions Pain**				
POD1	16.0 (15.0–17.0)	17.0 (16.0–18.0)	-1 (-2 to 0)	0.027*
POD2	18.0 (17.0–19.0)	19.0 (18.0–20.0)	-1 (-2 to 0)	0.024*
**Physical comfort**				
POD1	38.0 (35.0–40.0)	39.0 (36.0–42.5)	-2 (-4 to 0)	0.041*
POD2	45.0 (42.0–47.0)	47.0 (45.0–50.0)	-2 (-4 to -1)	0.005*
**Physical independence**				
POD1	17.0 (15.0–18.0)	17.0 (17.0–18.0)	-1 (-1 to 0)	0.061
POD2	18.0 (18.0–19.0)	18.5 (17.0–19.5)	0 (-1 to 0)	0.381
**Physical support**				
POD1	17.0 (16.0–18.0)	17.0 (16.0–18.0)	0 (-1 to 1)	0.762
POD2	18.0 (17.0–20.0)	19.0 (17.0–19.5)	0 (-1 to 1)	0.789
**Emotional state**				
POD1	33.0 (31.0–34.0)	35.0 (31.5–35.0)	-2 (-3 to 0)	0.018*
POD2	34.0 (32.0–37.0)	38.0 (36.0–39.0)	-3 (-4 to -1)	0.000*
				0.789 0.018* 0.000*

Data are presented as median (IQR). The median difference (reported with 95% CI) is the median of all pairwise differences between observations in the two groups. It is not the difference between the group medians. *P < 0.05, compared with control group. Abbreviations: CI: confidence interval; IQR, interquartile range; QoR-15, 15-item quality of recovery; POD1, Postoperative day 1; POD2, Postoperative day 2

Cognitive status was determined using MMSE, which was assessed at Pre, POD1, and POD2. In present study, the MMSE scores both at Pre and POD2 showed no statistically difference between the two groups (*P* > 0.05). However, at POD1, there was a significant difference in MMSE scores between the S-ketamine and control groups, and the scores in S-ketamine group were higher than the control group (*P* < 0.05). These results demonstrated that general anesthesia combined with S-ketamine was more propitious to facilitating early postoperative cognitive function rehabilitation of breast cancer patients (Fig. 2B).

**Fig. 3:**
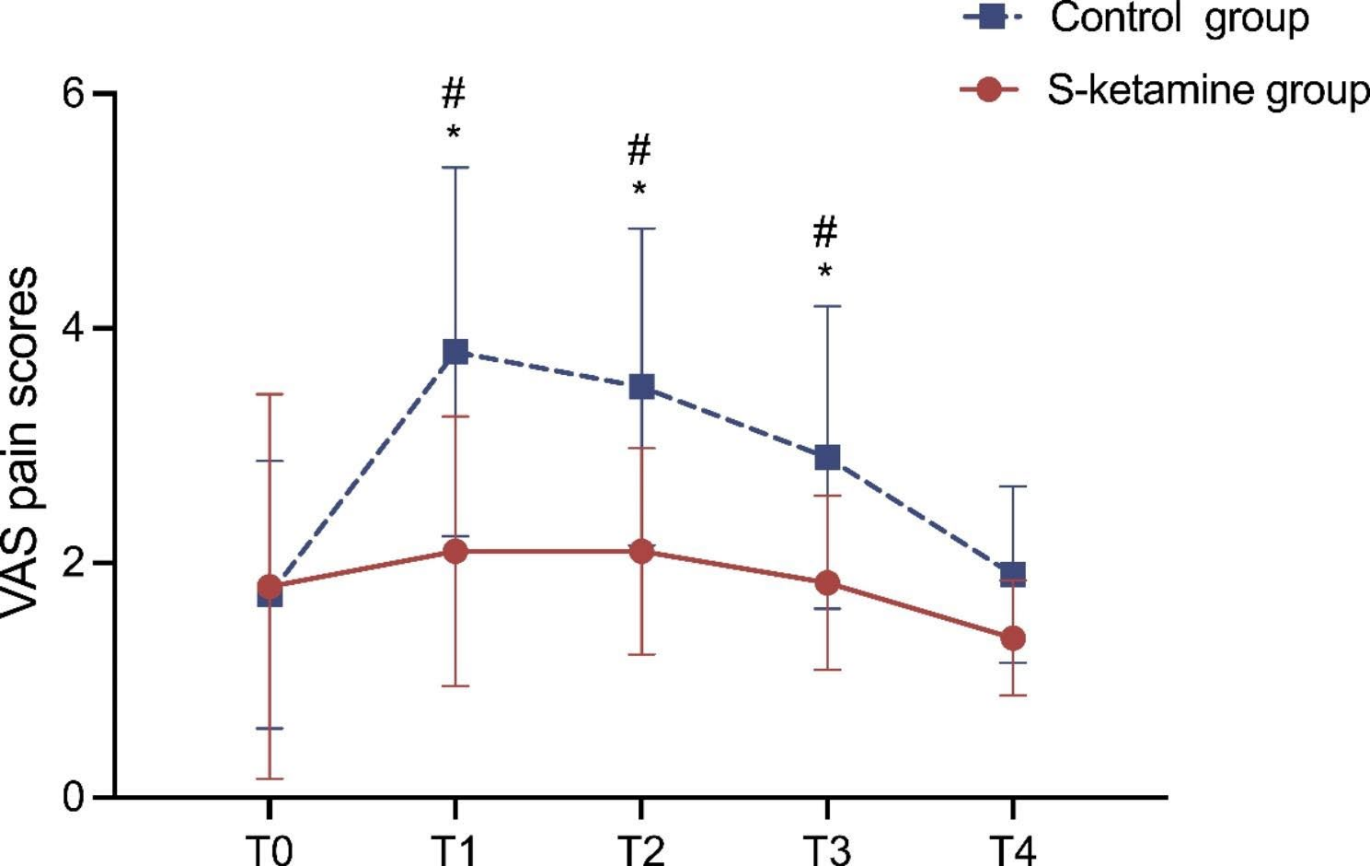
The comparison of the VAS scores of patients in two groups. ^#^*P* < 0.05, compared with T0. *P < 0.05, compared with control group. Abbreviations: VAS, Visual Analogic Scale; T0, The end of surgery; T1, 4 h after surgery; T2, 6 h after surgery; T3, 24 h after surgery; T4, 48 h after surgery

Postoperative pain intensity of the participants illustrated in Fig. 3. Notably, VAS scores assessed at T0 and T4 were similar between the two groups. The VAS score at 4 h, 6 h, and 24 h after surgery was significantly lower in the S-ketamine group than those in the control group (*P* < 0.05). The current results indicated that S-ketamine has great potential for perioperative and postoperative analgesia, and it can effectively reduce the intensity of postoperative pain, which means that S-ketamine for perioperative analgesia is a promising option (Fig. 3).

The perioperative adverse reactions and patient’s satisfaction in the two groups are shown in Table 3. Non-significant differences were observed in terms of the duration of PACU and occurrence of dizziness and agitation between the two groups (*P* > 0.05). None of the patients exhibited any of the psychotomimetic adverse reactions associated with S-ketamine, such as hallucinations or nightmares. The incidence of PONV in the control group was 27.5% (11/40), which was significantly higher when compared with the S-ketamine group (7.5%, 3/40) (*P* = 0.019). Furthermore, compared with control group, the anesthesia satisfaction score was significantly increased in the S-ketamine group. And it is worth to note that the application of remedial analgesia within 48 h was significantly lower in the S-ketamine group when compared with the control group (2 [5.0%] vs. 9 [22.5%]; *P* < 0.05). While there were no differences with respect to the number of patients requiring remedial analgesia while in the PACU between control and S-ketamine group (3 vs. 0, *P* > 0.05) (Table 3). These results suggested that S-ketamine could reduce the application of rescue analgesia, incidence of PONV, and improve patient’s satisfaction within 48 h after surgery and can be efficiently carried out in patients with BC without increasing the incidence of complications.

**Table 3:** Adverse events in the perioperative period (n = 40 in each group)

	Control Group	S-ketamine Group	*t /Z /X2*	*P*-value
Length of PACU stay (min)	38.0 (30.0–40.0)	35.0 (30.0–40.0)	-0.353	0.726
Occurrence of PONV	11.0 (27.5%)	3.0 (7.5%)	5.541	0.019*
Occurrence of dizziness	3.0 (7.5%)	3.0 (7.5%)	0.000	1.000
Occurrence of agitation	6.0 (15.0%)	4.0 (10.0%)	0.457	0.499
Remedial analgesia	9.0 (22.5%)	2.0 (5.0%)	5.165	0.023*
Patient satisfaction score	8.0 (8.0–9.0)	9.0 (9.0–10.0)	4.959	< 0.001*

Data are presented as median (IQR) or number (percentage). *P < 0.05, compared with control group. Abbreviations: PACU, Post-anesthesia care unit; PONV, Postoperative nausea and vomiting

## Discussion

In order to explore the potential effects of general anesthesia combined with S-ketamine on postoperative quality of recovery (QoR-15) and cognitive function in patients undergoing MRM. In this study, patients with BC were allocated to either the S-ketamine or control intervention group. Our results revealed that general anesthesia with S-ketamine can improve the early QoR-15 score both at POD1 and POD2. When assessed with regard to MMSE score, we have observed that the score measured in the S-ketamine group had a higher MMSE score than in the control group on POD1, but not on POD2. Furthermore, we confirmed that general anesthesia with S-ketamine brings substantial favorable benefits to postoperative pain management, decreases the opioid and propofol consumption. We believe that the findings of this study are of clinical guiding significance to evaluate general anesthesia with S-ketamine for BC patients from the perspective of postoperative recovery.

Recovery from anesthesia and surgery is a complicated process and maybe affected by various factors. Poor recovery may increase the likelihood of postoperative morbidity. Therefore, improving postoperative rehabilitation is of great significance to surgery patients. The QoR-15 is one of the most frequently used, well-known patient-reported scale [16], which has been confirmed validity and reliability in assessing the quality of a patient’s recovery from anesthesia [17]. In addition, research has shown that the early QoR-15 score after anesthesia has proved that it has predictive validity, which was moderately related to the incidence of postoperative adverse outcomes [18]. Collectively, it is reliable and comprehensive to use QoR-15 scale to evaluate the recovery from surgery and anesthesia.

In this study, the QoR-15 scale was applied to measure and compare the quality of postoperative rehabilitation between the two groups. The present results showed that S-ketamine can significantly improve the quality of recovery at POD1 and POD2. Moreover, further analysis conducted from the five subcomponents of QoR-15 manifested an increase of QoR-15 scores in the S-ketamine group was mainly reflect in pain, physical comfort, and emotional state, which account for 110 points out of the total score-pain (20 points), physical comfort (50 points) and emotional state (40 points). In other words, S-ketamine can effectively control patients’ postoperative pain, relieve their negative emotions, and improve patients’ physical comfort, thereby facilitating postoperative rehabilitation. Wang et al. confirmed that S-ketamine could improve short-term depression and recovery from anesthesia [19]. Another research has drawn the same conclusion that ketamine being identified as a portion of an enhanced recovery program for patients undergoing bariatric surgery [9]. Nevertheless, we did not find any improvements with regard to psychological support score and physical independence score regarding the S-ketamine intervention. We speculated that this discrepancy might be attributed to the following reasons: First, our study included a younger population, who generally have better psychological support and physical independence. Second, the patients we enrolled belongs to ASA grade I or II, with fewer comorbidities. Therefore, the benefit of S-ketamine in elderly patients and patients with more comorbidities on QoR should also be validated in future prospective clinical trials.

Based on previous studies and our results, we propose that the benefit of S-ketamine underlying this phenomenon is probably because of a combination of several factors: First, this maybe correlated with strong analgesic and antidepressant effects of S-ketamine, which could provide optimal postoperative pain management and alleviate the anxiety and depression caused by disease and surgical trauma. Second, it may be significantly related to the reduction of the occurrence of postoperative remedial analgesia and PONV, which may strengthen patient’s physical comfort and satisfaction.

MMSE is the most commonly used cognitive functional assessment scale with many advantages, including easy evaluation and high acceptability by both doctors and patients [20]. The risk of cognitive dysfunction has been associated with several perioperative factors, for example, anesthesia, surgery, mental disorders, and perioperative inflammatory response, etc. Herein, we found that general anesthesia combined with S-ketamine could promote the recovery of postoperative cognitive function on POD1, which was consistent with Tu et al’s results [7]. And similar conclusions were reached in a clinical study of S-ketamine, it was observed that the serum concentration of S-100β and MMSE scores in the S-ketamine group was lower than that in the control group at 24 h after surgery [21]. We believed that the effect of cognitive function improvement of S-Ketamine in this study may be related to the increase of cerebral blood flow and the decrease of excitatory transmitter glutamate concentration and inflammatory response. But, on POD2, no marked difference was observed in MMSE score between two groups; this may be correlated with a relatively young age enrolled in this trial, for several researches confirmed that age is an independent risk factor for postoperative cognitive dysfunction. It is noteworthy that other relevant studies indicated that S-ketamine was associated with cognitive performance decline [22], which was contrary to our results. Therefore, the protective effect of S-ketamine against postoperative cognitive impairment needs further investigation. In addition, in this study, we failed to observe that anesthesia induction with S-ketamine alone also has a related improvement in postoperative cognitive function. And future studies should examine in animals whether induction of S-ketamine alone is associated with improved cognitive function after surgery.

At present, the specific mechanism of S-ketamine to improve postoperative cognitive dysfunction remains to be further studied. Existing studies have shown that S-ketamine plays an important role in improving neuroinflammation. Wang et al. found [23] that S-ketamine can reduce the level of inflammatory factors by inhibiting TLR4/NF-κB signaling pathway of microglia cells in the central nervous system of aging mice, improve neuroinflammatory response, and then relieve postoperative cognitive dysfunction. In addition, Niu et al. found that [24] preoperative administration of a subanesthetic dose of ketamine activated Bmal1 mRNA expression and down-regulated the NMDA/NF-κB axis in elderly mice undergoing partial hepatectomy. And reduced inflammation, thereby alleviating postoperative cognitive decline in mice. Although the specific mechanism of S-ketamine improving POCD is still only studied in the classical inflammatory signaling pathway such as NF-κB, we have reason to believe that S-ketamine is likely to relieve postoperative cognitive dysfunction by reducing neuroinflammation.

Especially, intensive perioperative analgesia management is essential to accelerate functional recovery, minimize discomfort, decrease the side effects and to increase overall patient satisfaction. Even the widely used, a great deal of researches confirmed that classic opioid medications are closely related to undesirable adverse reactions such as PONV, respiratory depression, cognitive impairment, ileus, urinary retention, and opioid-induced hyperalgesia [25]. There is no doubt that these adverse reactions may inhibit postoperative functional recovery, reduce patient satisfaction, and increase the possibility of postoperative chronic pain [26]. Additionally, the findings of a meta-analysis revealed that, perioperative S-ketamine administration is a potent adjunct to pain management, which leads to a reduction in opioid consumption, hospital stay and hastens recovery of anesthesia in patients following major surgery [27]. In the present study, 0.5 mg/kg S-ketamine was used instead of sufentanil for anesthesia induction, together with remifentanil for anesthesia maintenance. This produces both current and long-term benefits to cancer patients, such as stabilized blood pressure, fewer opioids consumption, minimized opioid-related side effects, better postoperative pain control, and fewer requirement of additional remedial analgesia, which was correlated with enhanced quality of postoperative functional recovery. This result was in accordance with several studies, which also verified that S-ketamine administration was effective for assisting analgesia [28]. Optimal perioperative management is desirable in patients undergoing surgical procedures. However, the selection of optimal analgesia for general anesthesia is still challenging, considering that we found that S-ketamine could not completely replace opioids, although it was a good alternative as an adjunctive analgesic.

Our study also indicated that the safety profile of S-ketamine was promising. The most common adverse events of S-ketamine include dissociation, nausea, vertigo, dysgeusia, dizziness, and psychomimetic side effects [12]. Our study indicated that there was no statistical significance in the occurrence of dizziness and agitation, and none of patients presented any other complaints attributable to S-ketamine. In addition, we observed significant differences in the incidence of opioid-related adverse events (nausea, vomiting and inadequate analgesia) across groups, which maybe reflect the benefits of opioid-sparing effects. This result consistent with Brinck et al’s research [29], but in contrast to other studies, in which S-ketamine did not decrease the rate of PONV [30]. Therefore, multiple clinical trials need to be conducted to verify this conclusion. Existing studies have suggested that sub-anesthetic doses of S-ketamine can alleviate postoperative pain but delay anesthetic recovery during laparoscopic cholecystectomy [31]. Differs from Zhang et al’s studies, no significant difference was observed with respect to the PACU recovery time between the S-ketamine and control groups. Additionally, one interesting thing presented in this work was that the patient satisfaction scores was significantly higher in the S-ketamine group than that of the control group. We believed that this phenomenon might be because of the administration of S-ketamine, given that it could enhance the postoperative analgesic effect, improve patients’ discomfort, relieve anxiety and depression of patients, and have less adverse reactions, thereby being more conducive to promote patient satisfaction.

Nevertheless, it should be acknowledged that there are various limitations in this trial. First, this study only evaluated the QoR-15, MMSE, and pain score within 48 h after surgery. We did not carry out follow-ups regarding the long-term quality of life, cognitive function, and chronic pain after surgery. Therefore, understanding the effects of S-ketamine on long-term cognitive function, functional recovery, and chronic pain in cancer patients is of great research value. Second, the emotional recovery of patients was assessed only using the QoR-15 scale. Future studies with a more rigorous design are needed to assess the emotional recovery on a special depression assessment scale.

## Conclusions

Collectively, our findings support that general anesthesia with S-ketamine as a potential strategy showed high safety and can not only improve the quality of recovery mainly through improving physical comfort, pain, and emotional state but also promote the recovery of cognitive function on postoperative 1st day in patients following MRM without increasing the rate of adverse reactions.

## Data Availability

All data generated or analyzed during this study are included in this published article.

## Abbreviations

ASA: American Society of Anesthesiologists
BMI: Body Mass Index
QoR-15: Quality of Recovery-15
MMSE: Mini-Mental State Examination
PACU: Post-anesthesia care unit
PONV: Postoperative nausea and vomiting
VAS: Visual Analogic Scale
